# Hydrodynamic Behavior of the Intrinsically Disordered Potyvirus Protein VPg, of the Translation Initiation Factor eIF4E and of their Binary Complex

**DOI:** 10.3390/ijms20071794

**Published:** 2019-04-11

**Authors:** Jocelyne Walter, Amandine Barra, Bénédicte Doublet, Nicolas Céré, Justine Charon, Thierry Michon

**Affiliations:** UMR 1332 Biologie du Fruit et Pathologie, INRA, Université de Bordeaux, CS 20032, 33140 Villenave d’Ornon, France; amandine.barra@inra.fr (A.B.); benedicte.doublet@inra.fr (B.D.); nicolas.cere@hotmail.fr (N.C.); justine.charon@sydney.edu.au (J.C.)

**Keywords:** intrinsically disordered protein, plant virus, eIF4E, VPg, potyvirus, molten globule, protein-protein interaction, fluorescence anisotropy, protein hydrodynamics

## Abstract

Protein intrinsic disorder is involved in many biological processes and good experimental models are valuable to investigate its functions. The potyvirus genome-linked protein, VPg, displays many features of an intrinsically disordered protein. The virus cycle requires the formation of a complex between VPg and eIF4E, one of the host translation initiation factors. An in-depth characterization of the hydrodynamic properties of VPg, eIF4E, and of their binary complex VPg-eIF4E was carried out. Two complementary experimental approaches, size-exclusion chromatography and fluorescence anisotropy, which is more resolving and revealed especially suitable when protein concentration is the limiting factor, allowed to estimate monomers compaction upon complex formation. VPg possesses a high degree of hydration which is in agreement with its classification as a partially folded protein in between a molten and pre-molten globule. The natively disordered first 46 amino acids of eIF4E contribute to modulate the protein hydrodynamic properties. The addition of an N-ter His tag decreased the conformational entropy of this intrinsically disordered region. A comparative study between the two tagged and untagged proteins revealed the His tag contribution to proteins hydrodynamic behavior.

## 1. Introduction

Many biologically functional protein regions do not fold spontaneously. This class of proteins, termed intrinsically disordered proteins (IDP), contains intrinsically disordered regions (IDR) which are devoid of stable secondary and tertiary structures under physiological conditions and rather, exist as dynamic ensembles of inter-converting conformers [1]. Many of these proteins gain a stable 3D structure only when they interact with their target molecules [2]. The ability to exert specific biological functions and to interact with various partners in spite of the lack of a precise 3D scaffold, challenges the classic paradigm according to which specificity can only be achieved through surface complementation between structured and conserved domains. It is now well accepted that intrinsic disorder is involved in a large spectrum of functional properties modulated through multi-partnership interactions with proteins and nucleic acids. With between 7.3% and 77% of residues being disordered, the proteome of viruses on the whole presents the highest variability of intrinsic disorder in the living world [3,4,5]. The genus *Potyvirus* represents one of the largest and most economically damaging genus of plant-infecting viruses [6]. These viruses possess a single-stranded, polyadenylated, positive-sense genomic RNA which is covalently linked at its 5′ end to a viral protein, the viral protein genome-linked (VPg) [7,8]. The VPgs from several potyviruses, namely lettuce mosaic virus (LMV) [9], potato virus Y (PVY) [10], and Potato virus A (PVA) [11] have been experimentally characterized as intrinsically disordered. The potyviral VPg has been shown to interact with several viral and host factors. It is assumed to be a multifunctional protein involved in essential steps of the virus infectious cycle, translation, replication, and movement [12,13]. The VPg recruits the host eukaryotic translation initiation factor 4E (eIF4E), or its isoform eIF(iso)4E, in an interaction that is crucial for virus infection [14,15,16]. Mutations in the central region of VPg (residues 80–125) are associated with host resistance breakdown, [17,18,19]. In the LMV VPg, this central region interacts with eIF4E [20]. This region has been predicted to be an IDR for VPg from twelve potyviral species [9,21]. The study reported here analyzes the contribution of VPg and eIF4E flexible regions to the hydrodynamic properties of the two proteins, either monomeric, or associated in a binary complex.

## 2. Results and Discussion

### 2.1. Hydrodynamic Behavior of the Histidine Tagged Forms Assessed by Size Exclusion Chromatography

**Figure 1:**
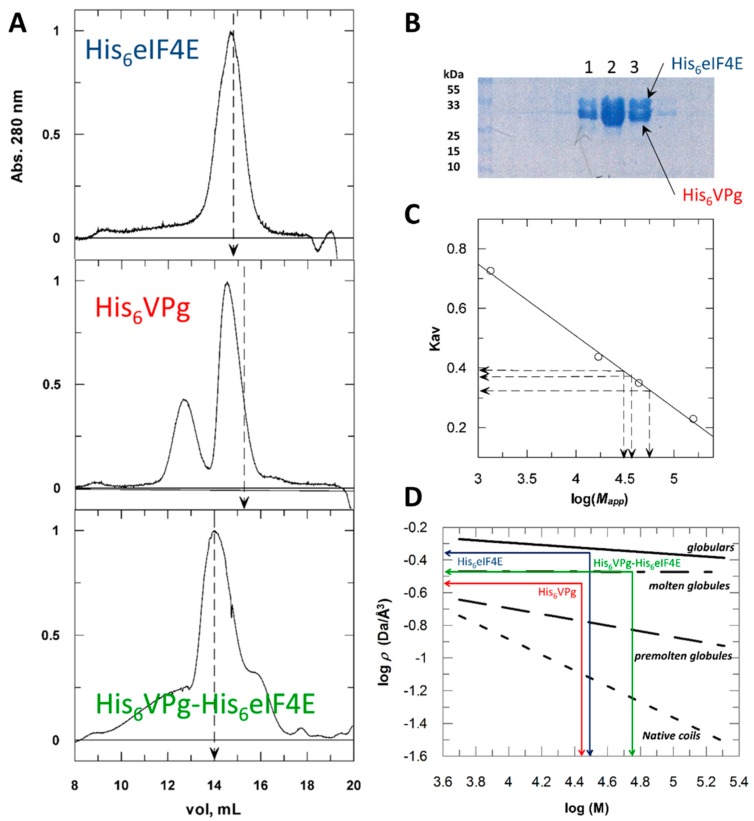
Size exclusion chromatography of His_6_ tagged VPg, His_6_ tagged 4E (eIF4E) and His_6_ VPg-His_6_ eIF4E, their binary complex. (**A**) Separated runs of the purified monomers (1 mL of 0.7–1 mg/mL) were performed. Vertical dashed lines refer to the elution volumes expected for globular proteins of 28,549 Da (upper panel) and 26,137 Da (middle panel), the molecular weights of recombinant His_6_ eIF4E and His_6_ VPg respectively (MALDI-TOF spectrometry determinations). The His_6_ VPg-His_6_ eIF4E binary complex was pre-formed by mixing His_6_ eIF4E (0.7 mg/mL with an excess of His_6_ VPg (1.2 mg/mL) and loaded up to the column (lower panel). A mass of 54,772 Da was estimated for the binary complex (summing His_6_ VPg and His_6_ eIF4E molecular weights) vertical dashed line. For comparison, absorbance values were standardized to the maximum value of each peak. (**B**) Distribution of the various molecular species through the size exclusion chromatography (SEC) separation of a His_6_ VPg-His_6_ eIF4E mix. Upon elution, fractions 1. 2 and 3 were recovered and submitted to SDS-PAGE analysis. (**C**) Determination of the three molecular species apparent molecular weights (*M_app_*) deduced from standard calibration with a set of known globular proteins. *K_av_* is a mean value determined from at least three independent SEC runs. (**D**) Apparent molecular densities (*ρ*) of the three molecular species were deduced from their experimentally determined hydrodynamic radius (*R_h_*) values (see material and methods). For each protein, the intersection between log*ρ* and log(*M*), *M* being the true molecular mass, allows to deduce the conformational families to which they belong to.

The secondary and tertiary structures of a protein involve non-covalent interactions. The more the amino-acid residues will interact, the more compact (globular) the protein will be. IDRs will involve fewer interactions between residues and lower compaction. Consequently, the hydration sphere of an intrinsically disordered protein is often larger than what would be expected for globular proteins of a similar molecular weight. A simple way to assess the degree of compactness of a protein in solution is to measure its hydrodynamic radius (*R_h_*). A commonly used method for measuring *R_h_* is size exclusion chromatography, SEC (Figure 1).

When submitted to SEC (Figure 1A), the hydrodynamic behavior of His_6_ eIF4E, *M* 28,550 Da (MALDI-TOF spectrometry of the purified recombinant protein), was in agreement with that of a 30 kDa globular protein. *R_h_* values were deduced from experimentally determined apparent molecular weights (*M_app_*) (see Section 4). The elution profile of purified recombinant His_6_ VPg, *M* 26,250 Da (mass spectrometry), featured two populations, with the major one (69%) suggesting that of a 40 kDa globular protein, and the minor species (27%) averaging 90 kDa. Clearly, His_6_ VPg did not behave like a globular protein. The trypsin hydrolysis kinetics of His_6_ VPg shows a moderate proteolytic resistance profile comparable to that of α-casein, a disordered protein (Figure S1). A method was developed to classify IDPs according to the relationship between their apparent molecular density (*ρ*) and their true molecular weight (*M*) [22]. From Figure 1C,D it can be deduced that His_6_ VPg shares hydrodynamic features of molten and pre-molten globules. The binary complex His_6_ VPg-His_6_ eIF4E showed a more complex elution profile (Figure 1A). Its major component (peak 2) displayed a *M_app_* of 56kDa, which is close to the weight (*M* 54,772 Da) calculated by adding the two partners molecular weights. SDS-PAGE analysis showed that this elution fraction contained equal amounts of His_6_ VPg and His_6_ eIF4E indicating a possible compaction of VPg upon its association with eIF4E. The major component eluted under peak 1, *M_app_* = 110 kDa, corresponds to oligomeric forms of His_6_ VPg (Figure 1B). The VPg propensity to aggregate was previously described [10].

Using PONDR-VLXT, intrinsic disorder was predicted for the tagged and untagged proteins. The His_6_ tag potentially brings disorder to the N-terminus of all proteins. After the His_6_ tag removal, the first 46 amino acids segment of the native eIF4E was still predicted as unstructured. This prediction was validated by previously reported structural data showing that the first 40 amino acids of eIF4E were intrinsically disordered and fold upon binding with eIF4G [23]. In addition, upon His_6_ tag removal, the first 25 amino acids of VPg were predicted as disordered (Figure S2). This region was recently reported as being a conformational switch [24]. Therefore, the contribution of these regions, predicted as disordered, to the hydrodynamic properties of the proteins was assessed.

The hydrodynamic behavior of VPg, eIF4E and their binary complex was deduced from fluorescence anisotropy measurements.

Because SEC leads to proteins diluting and to complexes partly dissociating during the chromatography process, it required substantial amount of proteins at concentration above 0.5 mg/mL. We experienced difficulties to obtain isolated untagged VPg and eIF4E at the concentrations suited for SEC experiments. Indeed, in vitro enzymatic tag cleavage resulted in a mixture of molecular species, which after separation (0.1–0.2 mg/mL), were not concentrated enough for SEC. Consequently, hydrodynamic parameters were deduced from fluorescence anisotropy measurements, which return a more detailed analysis of hydrodynamic behavior and can be operated at much lower concentrations. Anisotropy measurements give access to the rotational correlation time (*θ*) of the proteins. This parameter is strongly related to their hydrodynamic properties, as it depends on the protein shape and it is linked to the effective solvent shell accompanying the protein rotational diffusion. For that purpose, a fluorescent probe, *N*-acetyl-*N*′-(5-sulfo-1-naphtyl)ethylenediamine (AEDANS) was linked to the VPg single cysteine with an efficiency of 0.9 AEDANS moiety per VPg. In another set of experiments, AEDANS was also coupled to His_6_ eIF4E, eIF4E and eIF4E^Δ1–46^ to evaluate the first 1–46 disordered residues contribution to eIF4E compaction and also, more generally, the effect of the His_6_ tag on the compaction of monomeric forms. There are four cysteine residues within lettuce eIF4E, among which two are strictly conserved in plant orthologues. The modification resulted in a mean of 1.7 AEDANS moieties per molecule. The addition of the fluorophore did not alter the binding properties of His_6_ eIF4E, eIF4E, and eIF4E^Δ1–46^ to VPg (Figure S3). This result was not surprising as wheat eIF4E, either reduced, oxidized, or with a cysteine-to-serine mutation do not undergo structural changes and are functional, all binding m7GTP in a similar and labile manner [25]. In addition, in the structure of pea eIF4E, the two sulfur atoms are in close proximity but are clearly not bridged [26].

### 2.2. His Tagging Modulates Proteins Hydrodynamic Parameters

**Figure 2:**
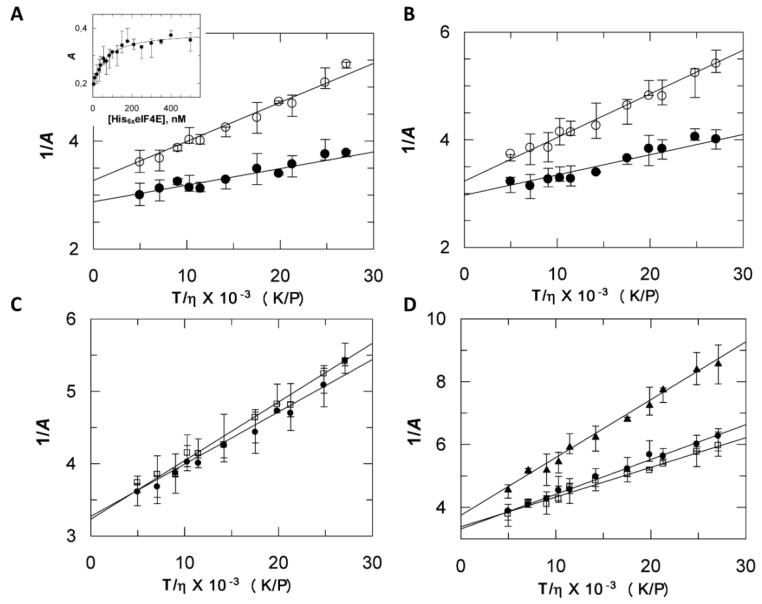
Fluorescence anisotropy of the various molecular forms of eIF4E and VPg. (**A**) The fluorescence anisotropy of His_6_ VPg* (300 nM, open circles) and His_6_ VPg*-His_6_ eIF4E complex (mix of 300 nM His_6_ VPg* and 2 µM His_6_ eIF4E filled circles) was recorded as a function of the viscosity increase at 25 °C. The reciprocal of the emitted light anisotropy (Perrin’s plot), 1/*A*, is plotted as a function of T/*η*. *V*, the apparent molecular volume of the proteins and their complexes and *A*_0_, the fundamental anisotropy were obtained respectively from the slope and intercept at infinite viscosity. Inset, the fluorescence anisotropy increase upon association of His_6_ eIF4E with His_6_ VPg* (300 nM). (**B**) Perrin’s plots of untagged VPg* (open circles) and VPg*-eIF4E complex (filled circles) in the same conditions as (**A**). (**C**) Perrin’s plots of His_6_ VPg* (open squares), VPg* (filled circles) and D. His_6_ eIF4E* (filled circles), eIF4E* (open squared) and eIF4E^Δ1–46^* (filled triangles). Experimental conditions were the same as in B. All measurements were obtained using VPg from lettuce mosaic virus (LMV) AF199 strain.

As expected, upon addition of His_6_ eIF4E, the fluorescence anisotropy of His_6_ VPg*, increased proportionally to the amount of His_6_ VPg*-His_6_ eIF4E complex formed. It reached a plateau value indicating a saturation, Figure 2A inset.

The modification by the probe did not significantly change the binding strength as comparable dissociation constants (*K*_D_) values were found for probed and unprobed proteins. A *K*_D_ value of 63 nM could be extracted from the data, in agreement with intrinsic fluorescence measurements (Figure S3). The hydrodynamic molar volume (*V*) and the rotational correlation time (*θ_exp_*) of the various molecular species were determined from steady state fluorescence anisotropy measurements as described in the experimental procedure section.

### 2.3. Discrepancies between SEC and Fluorescence Anisotropy Suggest a Contribution of Tags in Proteins Hydrodynamic Behavior

The parameters *V* and *θ_exp_* were derived for the various monomeric species (Figure 2C,D, and Table 1). The more the ratio *θ_exp_*/*θ_calc_* differs from unit, the more asymmetric the protein is. The *θ_exp_*/*θ_calc_* ratio of His_6_ VPg (2.41) and His_6_ eIF4E (1.62) indicated that their shape differed from a sphere. In addition, although the His_6_ VPg molecular weight was 4295 Da less than His_6_ eIF4E, its *V* value was 1.37 times larger, indicating that it was significantly less compact, a pre-molten globule feature. The untagged form of VPg displayed an expected decrease of its hydrodynamic molar volume with respect to its tagged form. Interestingly, the eIF4E* untagged form showed a higher *V* value (46.5 L/mol) and hence, a significant decrease in compaction compared to His_6_ eIF4E* (42.9 L/mol). This could be due to interactions between the His_6_ tag and the eIF4E N-ter IDR [23], an effect previously discussed [27]. Because of the intrinsic conformational entropy and the positive charges cluster of the disordered His_6_ tag, its interactions with other parts of the protein are more likely to occur. This could account for the more compact hydrodynamic behavior of His_6_ eIF4E. For most 3D structures solved from tagged proteins, the peptide tag is too disordered to be resolved. However, it is worth mentioning that, in most of the cases, comparisons of tagged with corresponding untagged structures determined from X ray diffraction data revealed only minor structural differences of the type that might be observed when comparing two identical sequences solved in different space groups [28]. This tends to show that these purification tags generally had no significant effect on the structure of the native protein. However, the importance of the proteins hydrodynamics properties cannot be understated as it accounts for the polypeptide chain dynamics, which drives most of biological functions. This is exemplified through the richness of the functional interpretations provided by NMR data related to disordered segments [29].

A comparison of *V* values between tagged and untagged species allows for the discussing of tag contribution to compaction. A decrease of the Perrin’s plot slope was observed for His_6_ VPg*-His_6_ eIF4E binary complex when compared to the slope of His_6_ VPg* (Figure 2A). Upon analysis of this data, it was shown that the hydrodynamic molar volume *V* linked to the labeled VPg enlarged from 58 L/mol to 121 L/mol, (Table 1). The later, attributed to the complex, was 20% larger than could be expected by adding the *V* value of His_6_ VPg* and His_6_ eIF4E monomers.

Hence, the anisotropy approach revealed a possible contribution of the two flexible His tags conformational entropy to the proteins hydrodynamic behavior. By contrast, the *V* value experimentally determined for the native untagged binary complex was close to that of the sum of the monomer values (Table 1). Compaction can be estimated by *ρ*, the molecular density value. Interestingly, the compaction within the binary complex suggested by SEC was not observed by anisotropy. In the SEC experiments, elution is concomitant to dilution, and hence to a modification of the species distribution. This could contribute to average the observed apparent molecular weight of each species. As stated in the experimental procedures section, anisotropy measurements on binary complexes as a function of various viscosity values were performed after the molecular species in presence have reached an equilibrium. The *ρ* value is directly obtained from *V* the experimental molecular volume, and thus, the anisotropy approach provides a likely more accurate way to estimate compaction than SEC does through the use of *M_app_* in the empirical linear Equation (4). More generally, *ρ* values provided by anisotropy were 1.5 time higher than those deduced from the SEC (Table 1).

### 2.4. His_6_ VPg* and His_6_ VPg*-His_6_ eIF4E Binary Complex Shapes Differ from the Globular State

The deduced experimental rotational correlation time, *θ_exp_*, increased from 23.7 ns for His_6_ VPg* to 49 ns for the His_6_ VPg*-His_6_ eIF4E binary complex. The *θ_calc_* values for hydrated rigid spheres of 26 kDa and 54.7 kDa were 9.8 ns and 20.6 ns (His_6_ VPg* and His_6_ VPg*-His_6_ eIF4E respectively), indicating that His_6_ VPg* and His_6_ VPg*-His_6_ eIF4E shapes differ from the compact globular state. On an indicative basis, the compact EΔ(1–46) [26] displayed an *θ_exp_* value (7.9 ns) which was close to the *θ_calc_* value (9.8 ns) calculated for a globular protein of comparable molecular weight. The *θ_exp_*/*θ_calc_* ratio for the His_6_ VPg*-His_6_ eIF4E complex and His_6_ VPg* were close, suggesting that the complex formation has no effect on the probe segmental motion within the VPg [30].

### 2.5. Compaction and Hydration are Experimentally Correlated

Because of their non-compact structures and more solvent-accessible surface area, disordered regions tend to display a higher hydration water density as compared to more ordered regions [31]. The degree of hydration (*h*) of each species was estimated from *θ_exp_* (see experimental section). For compact globular proteins, this value is usually in between 0.2 and 0.4 g water/g protein. Our data suggests that His_6_ VPg and VPg forms are more solvated than standard folded proteins (1.44 and 1.57 g/g respectively). This is in agreement with previous hydrodynamic experiments on IDPs. For instance, in the absence of calcium, the adenylate cyclase toxin calcium binding domain is intrinsically disordered and displays a high hydration propensity [32]. Moreover, molecular dynamics simulations show that partially disordered proteins like VPg have a higher capacity to bind hydration water as compared to globular proteins [33]. Interestingly, His_6_ eIF4E and eIF4E, which both include a long disordered N-terminal region, have a rather high hydration degree (0.72 and 1 g/g respectively) although the presence of the His_6_ tag seems to slightly decrease the hydration degree in accordance to its compacting effect discussed above. Conversely, eIF4E^Δ1–46^, which corresponds to the globular part of eIF4E, possesses a hydration value expected for compact proteins (0.38 g/g). Using the PDB coordinates of eIF4E^Δ1–46^ from pea (PDB file 2WMC), we calculated the protein rotational diffusion coefficient (*D*) [34] from Equation (12). From this value (1.49 × 10^7^ s^−1^) the rotational coefficient time was deduced and, in turn a *h* value of 0.53 g/g, which is in fairly good agreement with our estimation (Table 1). Finally, the binary complex formation is not associated with a significant modification of hydration.

## 3. Conclusions

As opposed to fully disordered proteins, which display a random coil state, VPg possesses a significant content in secondary structure. These more compact intermediates have led to the concept of molten globule (MG) and the somewhat less compact pre-molten globule (PMG) states. A MG is characterized by a large internal flexibility of its side chains and backbone, with a *R_h_* 1.5–2.0 times larger than that of globular proteins. As they are usually distributed in solutions between a limited number of conformers, these proteins prove to be more complex to analyze than fully disordered polypeptides. Modern NMR approaches provide an excellent way to study such proteins [35,36]. However the concentrations required [37], usually from 2 to 10 mg/mL, are far beyond what can be stabilized in solution in the present case. Because it enables the development of low-resolution structural models, taking into account the contribution of intrinsically disordered regions, a hydrodynamic analysis can provide useful data. Hydrodynamic parameters are usually assessed using SEC, AUC (analytical ultracentrifugation), and DLS (dynamic light scattering). However, these techniques also require protein concentrations above 1 mg/mL. We propose an elegant way to deal with especially difficult proteins. Although less used, fluorescence anisotropy can prove quite resolutive and especially suitable when protein concentration is the limiting factor. One can argue that this method gives access to the rotational mobility of the reporter fluorophore and not of the macromolecule itself. However, if, as it is the case here, the probe motion displays a low degree of freedom within the macromolecule, the extracted parameters reflect the hydrodynamic properties of the macromolecule well. The data obtained by SEC and fluorescence spectroscopy were in good agreement. Because analytical ultracentrifugation (AUC) is based on equilibrium and non-equilibrium thermodynamics, it is referred as a gold standard for characterizing the hydrodynamic properties. The expected sedimentation coefficients (*s*_20,*w*_) for the LMV VPg and lettuce eIF4E were derived from their experimentally measured diffusion coefficients (see Equation (13) in the experimental section). They were in agreement with values reported for their homologous counterparts PVY VPg (46.7% identity, 76.4% similar with LMV VPg) and human eIF4E (43.2% identity, 67.6% similarity with lettuce eIF4E), (Table 2). A fine analysis of potyviral VPg conformers distribution on the basis of their sedimentation properties was recently reported [24]. A *s*_20,*w*_ value of 3.2 Svedberg was determined for the major VPg molecular species (70%). [10]. This suggests that most of the VPg was present as a dimer. Indeed, this value is in accordance with the value obtained for non-reduced VPg from PVY [10], Table 2.

## 4. Materials and Methods

### 4.1. Protein Preparation

The gene coding for eIF4E initiation factor from the lettuce (Lactuca sativa cultivar Salinas GenBank AF 530162) and its derived molecular species were cloned into the vector pENTR/D-TOPO^®^ (Invitrogen, Carlsbad, CA, USA). They were transferred into pDEST^TM^17 using the Gateway^®^ recombinant Technology to allow production of N-terminal fusions with an hexahistidine tag (Invitrogen). In addition, full length eIF4E and eIF4E ^(Δ1–46)^ were cloned into the vector pENTR/SD/D-TOPO^®^ (Invitrogen) and transferred into the Gateway pDEST^TM^14 expression vector according to manufacturer’s instructions to obtain untagged full length eIF4E and eIF4E^(Δ1–46)^. The constructs were introduced into *E. coli* (BL21-AI strain), expression and purification of the His-tagged proteins were performed on ion metal affinity chromatography followed by m7GTP-Sepharose 4B (GE Healthcare, Amersham, UK) as previously described [39]. Untagged proteins were obtained by one step affinity purification on m7GTP sepharose 4B. The Lettuce mosaic virus VPg (isolate AF199GenBank AJ2 78854) coding sequence was cloned into the pTrcHis C expression vector downstream from an hexahistidine tag (Invitrogen). The vector contains a specific enterokinase cleavage site in frame with the protein for proteolytic tag removal. The protein was produced and purified as previously reported [39] except that the final monoQ chromatographic step was replaced by a size exclusion chromatography on a Superdex 75 HR 10/30 column (GE Healthcare) in 20 mM Hepes pH 8, 300 mM NaCl, and 2 mM DTT. For His-tag removal, this last step was omitted and the protein was diluted twice in the same buffer with reduced ionic strength (150 mM NaCl) containing 1 mM CaCl_2_, 0.1% Tween-20. His tagged enterokinase (0.02 mg for 0.2 mg VPg) was added and the protein mix was dialyzed overnight at 4 °C against the same buffer. The protease and uncleaved tagged VPg were subsequently trapped on a NiNTA resin and the pure free VPg form was recovered in the flow through (30–40% yield).

Protein labelling with *N*-(iodoacetyl)-*N*′-(5-sulfo-1-naphtyl)ethylenediamine, (IAEDANS).

Cysteine residues were reduced before labelling. The VPg or eIF4E solutions (0.2 mg/mL) were dialyzed overnight in 25 mM HEPES, pH 7.5, 6 mM βMe, 2 mM AcNa, 5 mM EDTA, 25 mM DTT, NaCl 0.3 M at 4 °C. The protein solutions (1 mL) were loaded on a 5 mL G25 column equilibrated in the coupling buffer (25 mM HEPES, pH 7.5, NaCl 0.3 M). A 10 times molar excess of IAEDANS was added in the same buffer and the mix was incubated in the dark at 25 °C for 2 h. The reaction was stopped by addition of DTT (25 mM final concentration). A buffer exchange was performed over a G25 equilibrated in 25 mM HEPES, pH 7.5, 150 mM NaCl. About 1 and 2 AEDANS molecules were bound per VPg and eIF4E respectively. 

### 4.2. Size Exclusion Chromatography

A superose 12 HR 10/30 column (GE Healthcare) was calibrated with separate 500 µL injections of the following native globular proteins: γ-globulin (bovine, 158 kDa), Ovalbumin (Chiken) 44 kDa, myoglobin (horse, 17 kDa and vitamin B_12_ (1.35 kDa). Excluded volume (*V*_0_, 8.74 mL) was determined with blue dextran. The volume of the column, *V*_t_ was 24 mL. Chromatography conditions were 20 mM HEPES/KOH, 0.4M KCl and 1.4 mM 2-Mercaptoethanol, flowrate 0.5 mL/min.

### 4.3. Hydrodynamic Radius Measure

The Stokes radius, also termed *R_h_*, is the radius of a hard sphere that diffuses at the same rate as the protein. A commonly used method for measuring *R_h_* is SEC. The size exclusion column is calibrated using the elution volume (*V_e_*) of standard folded proteins (i.e., Globular) of known molecular weight. The apparent molecular weight of the protein of interest is then deduced from *V_e_*. *R_h_* is determined as the *R_h_* expected for a globular protein of that apparent molecular weight, for which simple relations exist [22,40].

The retention factor *K_av_* of the proteins was determined as follows:

(1) $$K_{av}=\frac{V_{e}-V_{0}}{V_{t}-V_{0}}$$

There was a linear relationship between *K_av_* and log*M_app_* (Figure 1C):

(2) $$K_{av}=-0.24\log M_{app}+1.47$$

(3) $$M_{app}=10^{\frac{1.47-K_{av}}{0.24}}$$

It was assumed that the protein considered has the hydrodynamic behavior of its equivalent rigid sphere of *R_h_* and an *M_app_*. A linear relationship exists between log*M_app_* and log*R_h_* which, knowing the SEC derived apparent molecular weight of a protein, allows the determination of its *R_h_* [22]. For a globular protein, the expression is:

(4) $$\log\left(R_{h}\right)=-0.2+0.36\log\left(M_{app}\right)$$

### 4.4. Structural Feature Estimation

The apparent molecular density or compaction (*ρ*) of a globular protein is:

(5) $$\rho =\frac{M}{4/3\pi R_{h}^{3}}$$

with *M*, the molecular weight calculated from the protein amino acid composition. A plot of log(*ρ*) vs. log(*M*) allowed us to estimate the structural family; ordered globular, molten globule, pre-molten globule, or native coil-like protein (Figure 1D). Straight lines that define the different groups of conformational states were calculated from [41].

*Fluorescence measurements.* All steady state fluorescence acquisitions were obtained at 25 °C in 20 mM HEPES pH 7.5, 0.25 mM NaCl and 1 mM DTT using a SAFAS Xenius spectrofluorimeter (Monaco) equipped with a Peltier temperature controller. For optical characteristics of the instrument, see [39]. Affinity constants were deduced from steady state eIF4E tryptophan intrinsic fluorescence decrease upon titration by VPg as previously described [39]. In order to ensure that the system reached an equilibrium before measurements, a thorough mixing (gentle back and forth syringe flushing) of the various molecular species, was followed by a 10 min incubation. Then, an average value was collected during another 10 min, both for the acquisition of eIF4E steady state intrinsic fluorescence and anisotropy measurements.

Fluorescence anisotropy measurement. The fluorescent probe bound to the VPg was chosen so that *τ* its fluorescent lifetime be of the order of magnitude of *θ*, the rotational correlation time of the VPg in solution. *A,* the measured anisotropy is defined as:

(6) $$\frac{A_{0}}{A}=1+\frac{\tau }{\theta }$$

*A*_0_ is the fundamental anisotropy observed in the absence of other depolarizing processes such as rotational diffusion or energy transfer. If θ≫τ then the measured anisotropy is equal to *A*_0_ (infinite viscosity, no motion of the macromolecule). If θ≪τ then the anisotropy is zero. For the AEDANS group, a fluorescence lifetime of 15.6 ns was determined by phase-modulation fluorimetry on a SLM 4800 fluorimeter. This value is in the range of *θ* values for proteins (15–70 kDa).

The anisotropy of the AEDANS labelled VPg either free or associated with eIF4E was measured at 25 °C in solutions of various viscosity (*η*). The dependency of the anisotropy on viscosity is given by the Perrin equation:

(7) $$\frac{1}{A}=\frac{1}{A_{0}}+\alpha \frac{T}{\eta }$$

with

(8) $$\alpha =\frac{\tau R}{A_{0}V}$$

Plotting 1/*A* versus T/*η* gives usually a straight line. The viscosity was experimentally increased by addition of sucrose in the buffer. *V* the hydrodynamic molar volume was determined from *α,* the slope value:

(9) $$V=\frac{\tau R}{A_{0}\alpha }$$

The experimental rotational correlation time *θ_exp_* was deduced from *V*:

(10) $$\theta_{exp}=\frac{\eta V}{RT}$$

The rotational correlation time of an equivalent rigid sphere of the same molecular weight M was calculated as follows:

(11) $$\theta_{calc}=\frac{\eta M}{RT}\left(\bar{v}+h\right)$$

where $\bar{v}$ is the protein partial specific volume (usually 0.73 cm^3^/g) and h is the degree of hydration (g H_2_O per g of protein; usually 0.2 < *h* < 0.4); *R* = 8.31 × 10^7^ erg mol^−1^·K^−1^. From Equation (13), replacing the calculated rotational time by rotational correlation times experimentally determined for each molecular species, leads to an estimation of *h* their degree of hydration (Table 1). Alternatively, knowing *D*, the rotational diffusion coefficient of the protein, its rotational correlation time can be obtained:

(12) θ=16D

The expected sedimentation coefficient can be deduced as follows:

(13) $$s=\frac{MD\left(1-\rho_{20}\bar{v}\right)}{RT}$$

with *M*, molecular weight, *ρ*_20_ solvent density (water).

## Figures and Tables

**Table 1:** Physical parameters of the various molecular forms of VPg, eIF4E and of their binary complexes.

Parameter	Units	HV	V	HE	E	EΔ(1–46)	HVHE	VE
MW	g/mol	26,222 ^†^	21,781 ^†^	28,550 ^†^	26,076 ^†^	21,246 ^†^	54,772 ^‡^	47,857 ^‡^
**SEC**								
*V_R_*	mL	14.4	nd	14.8	nd	nd	13.7	nd
*M_app_*	kg/mol	38.0 ± 1.0	nd	30.2 ± 0.8	nd	nd	56.2 ± 1.5	nd
*R_h_*	Å	28.1 ± 0.7	nd	26.4 ± 1.1	nd	nd	33.0 ± 0.9	nd
*ρ*	g/mol·Å ^3^	0.28	nd	0.40	nd	nd	0.38	nd
Conformation		MG/PMG		F/MG			MG	
**^1^ Fluorescence Anisotropy**								
*V_app_*	L/mol	58.7 ± 1.8	51.6 ± 0.2	42.9 ± 0.8	46.5 ± 1.0	24.3 ± 0.5	121.3 ± 3.2	102.4 ± 0.9
*θ_exp_*	s (×10^9^)	23.7	20.9	17.3	18.8	9.8	49 ^3^	41.4
*θ_calc_*	s (×10^9^)	9.8	8.9	10.7	9.8	7.9	20.6	18.0
(*θ_exp_*)/(*θ_calc_*)		2.41	2.55	1.62	1.92	1.23	2.38	2.30
*h*	g water/g	1.44	1.57	0.72	1	0.38 (0.53) ^2^	1.41	1.35
*R_h_*	Å	24	23	22	22	18	31	29
*ρ*	g/mol·Å ^3^	0.45	0.42	0.67	0.56	0.88	0.45	0.47

HV, His_6_ VPg; HE, His_6_ eIF4E; V, His_6_ VPg; E, eIF4E; EΔ(1–46), 1–46 residues deleted eIF4E; HVHE, His_6_ VPg-His_6_ eIF4E binary complex; VE, VPg-eIF4E binary complex. MW (molecular weight), *V_R_* Retention volume), *M_app_* (apparent molecular mass), *R_h_* (hydrodynamic radius), *ρ* (apparent molecular density), *V_app_* (apparent molecular volume), *θ_exp_* (experimental rotational correlation time), *θ_calc_* (calculated rotational correlation time), *h* (estimated hydration degree). ^1^ Determined for *η* = 0.01 P (water) at 298 K; *τ*, AEDANS fluorescence lifetime: 15.6 ns; ^2^ Calculated from pea eIF4E structure (2WMC) using the Hydropro software (http://leonardo.inf.um.es/macromol/programs/hydropro/hydropro.htm); ^3^ value obtained from His_6_ VPg*-His_6_ eIF4E binary complex; ^†^ Mass spectrometry (MALDI-TOF); ^‡^ Calculated from amino acid sequence.

**Table 2:** VPgs and eIF4E sedimentation coefficients in water at 20 °C.

Molecular Species	MM, kDa *	*s*_20,*w*_, s (×10^13^)	Ref.	Method
VPg (LMV)-R	21.8	1.8	This study	Fluorescence
VPg (PVY)-R	22	1.7	[10]	UAC
VPg (PVY)	22	3.0	[10]	UAC
VPg (PVBV)	21.8	3.2	[24]	UAC
eIF4E (lettuce)	26.1	2.2	This study	Fluorescence
eIF4E (human)	24.3	2.03	[38]	UAC

LMV (lettuce mosaic virus), PVY (potato virus Y), PVBV (Pepper vein banding virus). * theoretical molecular weight. VPg (LMV)-R and VPg (PVY)-R, VPg under reduced conditions.

## Supplementary Materials

Supplementary materials can be found at https://www.mdpi.com/1422-0067/20/7/1794/s1.

## Abbreviations

LMV	Lettuce mosaic virus
PVY	Potato virus Y
TEV	Tobacco etch virus
TuMV	Turnip mosaic virus
IAEDANS	*N*-(iodoacetyl)-*N*′-(5-sulfo-1-naphtyl)ethylenediamine
AEDANS	*N*-(acetyl)-*N*′-(5-sulfo-1-naphtyl)ethylenediamine
His_6_ eIF4E	N-ter hexahistidine tagged lettuce eIF4E
His_6_ VPg	N-ter hexahistidine tagged LMV VPg
eIF4E^Δ1–46^	untagged lettuce eIF4E deleted from its first 46 N-ter amino acid
His_6_ VPg*	AEDANS labelled N-ter hexahistidine tagged LMV VPg
His_6_ eIF4E	AEDANS labelled N-ter hexahistidine tagged lettuce eIF4E
eIF4E*	AEDANS labelled untagged lettuce eIF4E
eIF4E^Δ1–46^*	AEDANS labelled untagged lettuce eIF4E deleted from its first 46 N-ter amino acids
[His_6_ VPg*-His_6_ eIF4E]	AEDANS labelled binary complex

## References

[1] Boehr D.D., Wright P.E. (2018). Biochemistry. How do proteins interact?. Science.

[2] Habchi J., Tompa P., Longhi S., Uversky V.N. (2014). Introducing Protein Intrinsic Disorder. Chem. Rev..

[3] Xue B., Blocquel D., Habchi J., Uversky A.V., Kurgan L., Uversky V.N., Longhi S. (2014). Structural Disorder in Viral Proteins. Chem. Rev..

[4] Pushker R., Mooney C., Davey N.E., Jacqué J.-M., Shields D.C. (2013). Marked Variability in the Extent of Protein Disorder within and between Viral Families. PLoS ONE.

[5] Xue B., Dunker A.K., Uversky V.N. (2012). Orderly order in protein intrinsic disorder distribution: Disorder in 3500 proteomes from viruses and the three domains of life. J. Biomol. Struct. Dyn..

[6] Adams M., Zerbini F., French R., Rabenstein F., Stenger D., Valkonen J. (2011). Potyviridae. Virus Taxonomy, 9th Report of the International Committee for Taxonomy of Viruses.

[7] Murphy J.F., Klein P.G., Hunt A.G., Shaw J.G. (1996). Replacement of the tyrosine residue that links a potyviral VPg to the viral RNA is lethal. Virology.

[8] Murphy J.F., Rychlik W., Rhoads R.E., Hunt A.G., Shaw J.G. (1991). A tyrosine residue in the small nuclear inclusion protein of tobacco vein mottling virus links the VPg to the viral RNA. J. Virol..

[9] Hebrard E., Bessin Y., Michon T., Longhi S., Uversky V.N., Delalande F., Van Dorsselaer A., Romero P., Walter J., Declerck N. (2009). Intrinsic disorder in Viral Proteins Genome-Linked: Experimental and predictive analyses. Virol. J..

[10] Grzela R., Szolajska E., Ebel C., Madern D., Favier A., Wojtal I., Zagorski W., Chroboczek J. (2008). Virulence Factor of Potato Virus Y, Genome-attached Terminal Protein VPg, Is a Highly Disordered Protein. J. Biol. Chem..

[11] Rantalainen K.I., Uversky V.N., Permi P., Kalkkinen N., Dunker A.K., Makinen K. (2008). Potato virus A genome-linked protein VPg is an intrinsically disordered molten globule-like protein with a hydrophobic core. Virology.

[12] Jiang J., Laliberte J.F. (2011). The genome-linked protein VPg of plant viruses-a protein with many partners. Curr. Opin. Virol..

[13] Martínez F., Rodrigo G., Aragonés V., Ruiz M., Lodewijk I., Fernández U., Elena S.F., Daròs J.-A. (2016). Interaction network of tobacco etch potyvirus NIa protein with the host proteome during infection. BMC Genom..

[14] Charron C., Nicolaï M., Gallois J.L., Robaglia C., Moury B., Palloix A., Caranta C. (2008). Natural variation and functional analyses provide evidence for co-evolution between plant eIF4E and potyviral VPg. Plant J..

[15] Leonard S., Plante D., Wittmann S., Daigneault N., Fortin M.G., Laliberte J.F. (2000). Complex formation between potyvirus VPg and translation eukaryotic initiation factor 4E correlates with virus infectivity. J. Virol..

[16] Robaglia C., Caranta C. (2006). Translation initiation factors: A weak link in plant RNA virus infection. Trends Plant Sci..

[17] Moury B., Charron C., Janzac B., Simon V., Gallois J.L., Palloix A., Caranta C. (2014). Evolution of plant eukaryotic initiation factor 4E (eIF4E) and potyvirus genome-linked protein (VPg): A game of mirrors impacting resistance spectrum and durability. Infect. Genet. Evol..

[18] Ayme V., Souche S., Caranta C., Jacquemond M., Chadoeuf J., Palloix A., Moury B. (2006). Different mutations in the genome-linked protein VPg of potato virus Y confer virulence on the pvr2(3) resistance in pepper. Mol. Plant Microbe Interact..

[19] Ayme V., Petit-Pierre J., Souche S., Palloix A., Moury B. (2007). Molecular dissection of the potato virus Y VPg virulence factor reveals complex adaptations to the pvr2 resistance allelic series in pepper. J. Gen. Virol..

[20] Roudet-Tavert G., Michon T., Walter J., Delaunay T., Redondo E., Le Gall O. (2007). Central domain of a potyvirus VPg is involved in the interaction with the host translation initiation factor eIF4E and the viral protein HcPro. J. Gen. Virol..

[21] Charon J., Theil S., Nicaise V., Michon T. (2016). Protein intrinsic disorder within the Potyvirus genus: From proteome-wide analysis to functional annotation. Mol. BioSyst..

[22] Uversky V.N. (2002). What does it mean to be natively unfolded?. Eur. J. Biochem..

[23] von der Haar T., Oku Y., Ptushkina M., Moerke N., Wagner G., Gross J.D., McCarthy J.E. (2006). Folding transitions during assembly of the eukaryotic mRNA cap-binding complex. J. Mol. Biol..

[24] Sabharwal P., Srinivas S., Savithri H.S. (2018). Mapping the domain of interaction of PVBV VPg with NIa-Pro: Role of N-terminal disordered region of VPg in the modulation of structure and function. Virology.

[25] Monzingo A.F., Dhaliwal S., Dutt-Chaudhuri A., Lyon A., Sadow J.H., Hoffman D.W., Robertus J.D., Browning K.S. (2007). The structure of eukaryotic translation initiation factor-4E from wheat reveals a novel disulfide bond. Plant Physiol..

[26] Ashby J.A., Stevenson C.E.M., Jarvis G.E., Lawson D.M., Maule A.J. (2011). Structure-based mutational analysis of eIF4E in relation to sbm1 resistance to Pea seed-borne mosaic virus in Pea. PLoS ONE.

[27] Marsh J.A., Forman-Kay J.D. (2010). Sequence determinants of compaction in intrinsically disordered proteins. Biophys. J..

[28] Carson M., Johnson D.H., McDonald H., Brouillette C., Delucas L.J. (2007). His-tag impact on structure. Acta Crystallogr. D Biol. Crystallogr..

[29] Mollica L., Bessa L.M., Hanoulle X., Jensen M.R., Blackledge M., Schneider R. (2016). Binding Mechanisms of Intrinsically Disordered Proteins: Theory, Simulation, and Experiment. Front. Mol. Biosci..

[30] Granon S., Kerfelec B., Chapus C. (1990). Spectrofluorimetric investigation of the interactions between the subunits of bovine pancreatic procarboxypeptidase A-S6. J. Biol. Chem..

[31] Aggarwal L., Biswas P. (2018). Hydration Water Distribution around Intrinsically Disordered Proteins. J. Phys. Chem. B..

[32] Chenal A., Guijarro J.I., Raynal B., Delepierre M., Ladant D. (2009). RTX calcium binding motifs are intrinsically disordered in the absence of calcium: Implication for protein secretion. J. Biol. Chem..

[33] Rani P., Biswas P. (2015). Local Structure and Dynamics of Hydration Water in Intrinsically Disordered Proteins. J. Phys. Chem. B..

[34] Ortega A., Amoros D., Garcia de la Torre J. (2011). Prediction of hydrodynamic and other solution properties of rigid proteins from atomic- and residue-level models. Biophys. J..

[35] Jensen M.R., Ruigrok R.W.H., Blackledge M. (2013). Describing intrinsically disordered proteins at atomic resolution by NMR. Curr. Opin. Struct. Biol..

[36] Wells M., Tidow H., Rutherford T.J., Markwick P., Jensen M.R., Mylonas E., Svergun D.I., Blackledge M., Fersht A.R. (2008). Structure of tumor suppressor p53 and its intrinsically disordered N-terminal transactivation domain. Proc. Natl. Acad. Sci. USA.

[37] Kosol S., Contreras-Martos S., Cedeño C., Tompa P. (2013). Structural characterization of intrinsically disordered proteins by NMR spectroscopy. Molecules.

[38] Modrak-Wojcik A., Gorka M., Niedzwiecka K., Zdanowski K., Zuberek J., Niedzwiecka A., Stolarski R. (2013). Eukaryotic translation initiation is controlled by cooperativity effects within ternary complexes of 4E-BP1, eIF4E, and the mRNA 5′ cap. FEBS Lett..

[39] Michon T., Estevez Y., Walter J., German-Retana S., Le Gall O. (2006). The potyviral virus genome-linked protein VPg forms a ternary complex with the eukaryotic initiation factors eIF4E and eIF4G and reduces eIF4E affinity for a mRNA cap analogue. FEBS J..

[40] Uversky V.N. (2002). Natively unfolded proteins: A point where biology waits for physics. Protein Sci..

[41] Uversky V.N., Santambrogio C., Brocca S., Grandori R. (2012). Length-dependent compaction of intrinsically disordered proteins. FEBS Lett..

