# Supplementary material for: Hydrodynamic Behavior of the Intrinsically Disordered Potyvirus Protein VPg, of the Translation Initiation Factor eIF4E and of their Binary Complex
Source: Int J Mol Sci. 2019 Apr 11;20(7):1794. doi: 10.3390/ijms20071794 (PMC6479716; doi:10.3390/ijms20071794)
Supplement: Supplementary file 1 [file ijms-20-01794-s001.pdf]

Hydrodynamic behavior of the intrinsically disordered potyvirus protein VPg, of the translation Initiation factor eIF4E and of their binary complex

Jocelyne Walter, Amandine Barra, Bénédicte Doublet, Nicolas Céré, Justine Charon and Thierry Michon*

UMR 1332 Biologie du Fruit et Pathologie, INRA-Université de Bordeaux, CS 20032, 33140 Villenave d'Ornon, France

Supporting Information

Figure S1: SDS-Page analysis of VPg proteolysis by trypsin limited digestion.

Figure S2: Disorder prediction for eIF4E and VPg.

Figure S3: Steady state titration of eIF4E-VPg binary complex formation

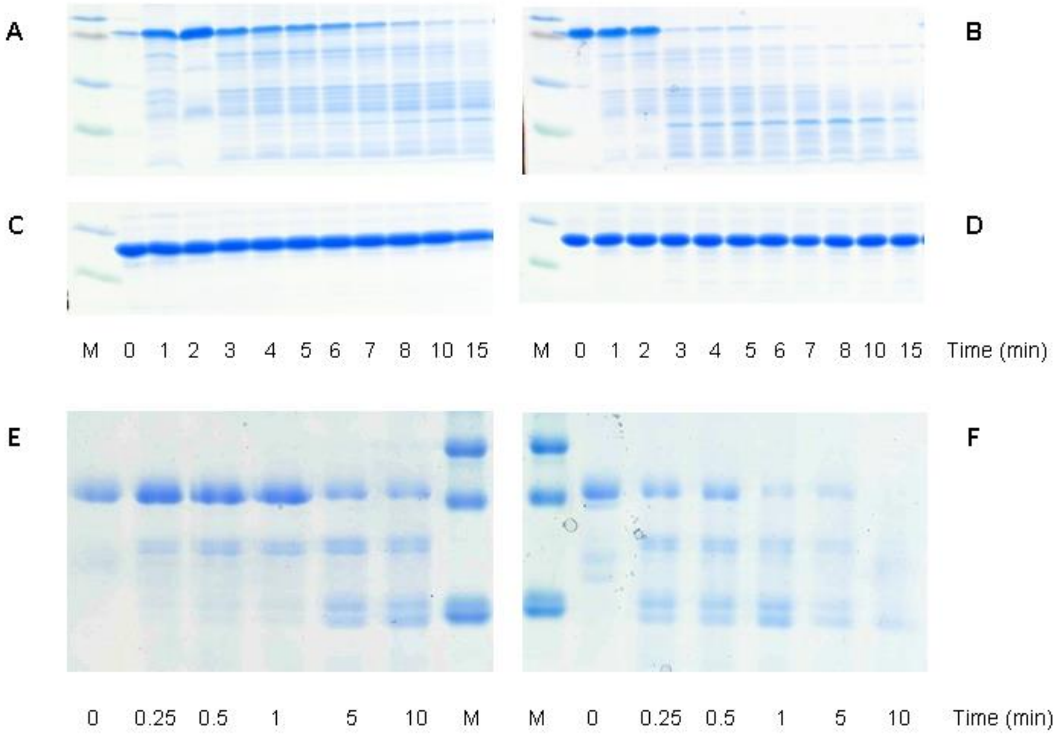

Figure S1: SDS-PAGE analysis of limited trypsin digestion of VPg and standard proteins by 0.2 µM trypsin (A,C,E) and 1 µM trypsin (B,D,F). Plots A and B show that α-casein, an intrinsically disordered protein is highly susceptible to digestion, whereas the highly ordered lysozyme is not (C and D). Plots (E and F), His_6-VPg digestion. The kinetics and profile of the VPg limited digestion at both 0.2 and 1 µM trypsin is typical of a protein containing disordered regions. Compared to α-casein, the VPg digestion pattern displays a moderate proteolytic resistance producing sizable stable fragments.

Contribution of disordered domains to the hydrodynamic behavior of VPg, eIF4E and their binary complex. In an attempt to predict the intrinsic disorder contain of the tagged ($(His)_6$VPg, $(His)_6$ eIF4E) and untagged deleted (eIF4E $^{[\Delta 1\text{-}46]}$) forms, we used PONDR-VLXT, a neural network predictor of natural protein disordered regions (1). This predictor is fed by DisProt, a curated database with proteins segments that lack fixed 3D structure in the Protein Data Bank (PDB) (2). PONDR-VLXT was chosen in this study, as its predictions are usually in good agreement with the disorder features observed in proteins showing larger hydrodynamic radi than expected by their molecular weight (3). In addition PONDR-VLXT predictions were in agreement with the experimental observation of disorder in VPgs from LMV, PVA and PVY. As shown on the prediction profile in figure 2, the hexahistidine tail potentially brings disorder to the N-terminus of all proteins. After the His_6 tag removal, the first 46 amino acids segment of the native eIF4E was still predicted as unstructured. This is in agreement with structural data showing that the first 40 amino acids of eIF4E constitute an intrinsically disordered domain. Upon His_6 tag removal, the first 25 amino-acids of VPg are predicted as disordered (figure S4).

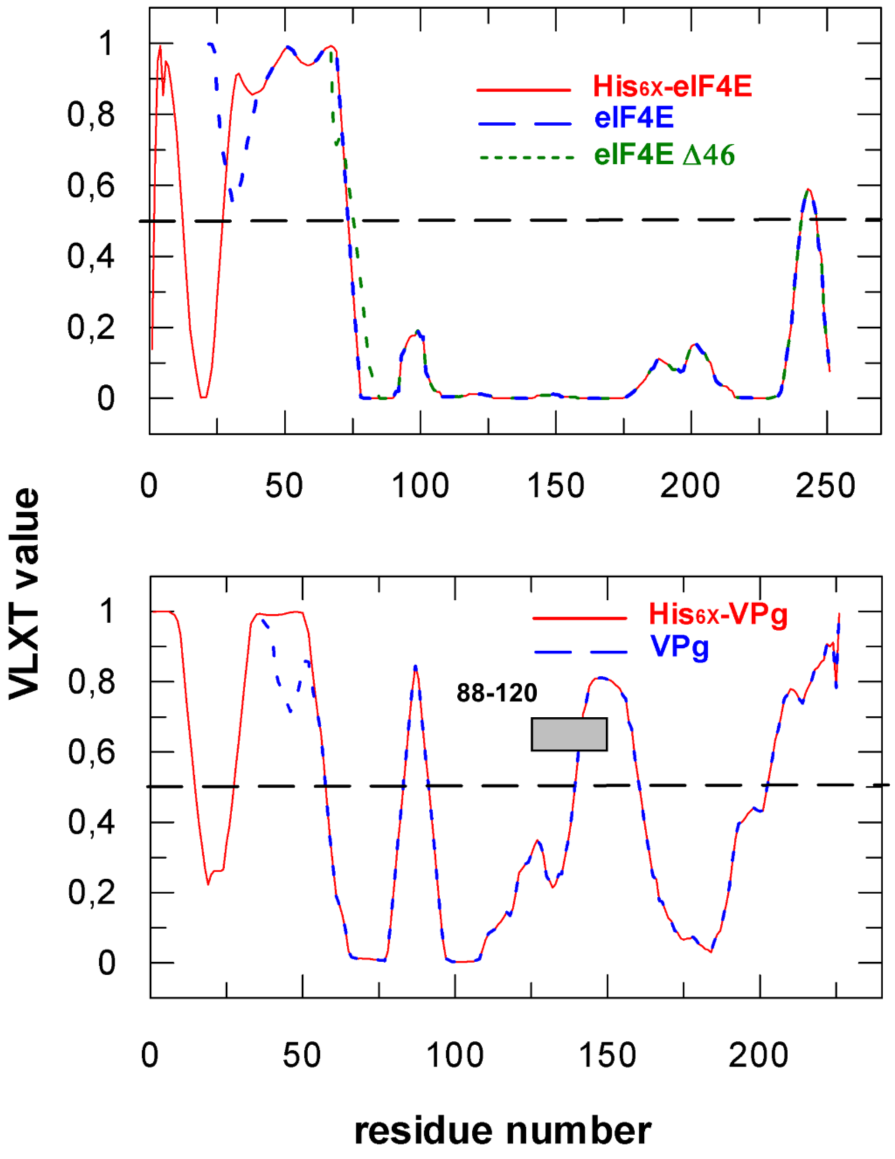

Figure S2. Disorder prediction for eIF4E and VPg, top and bottom panel respectively. Tagged forms (His_6-eIF4E and His_6-VPg), solid lines. Untagged forms (VPg and eIF4E), dashed lines, eIF4EΔ46 short form, dotted line. Above horizontal lines (score 0.5) domains are predicted as disordered. Grey box (88-120) refers to the position of the VPg_{89-105} peptide reported to interact with eIF4E (4). As residue numbering includes the His-tag, eIF4EΔ46 starts at position 74. The VPg_{88-120} peptide spans residue 126 to 158 in the tagged VPg. Predictions were run with PONDR-VLXT included in the PONDR-FIT package, (5).

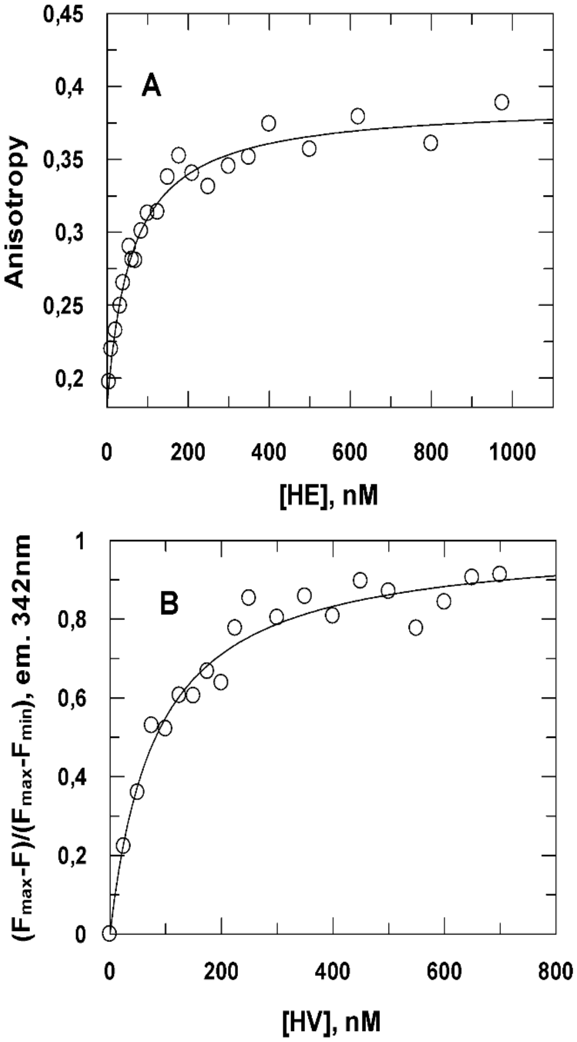

Figure S3: A. Fluorescence anisotropy increase accompanying VPg-eIF4E binary complex formation. His_6VPg was labeled with IAEDANS, (K_D=61±9 nM). Increasing amounts of eIF4E were added to 300nM VPg. B. steady state titration of lettuce eIF4E association with His_6VPg (K_D=82±10 nM). The decrease of eIF4E intrinsic fluorescence upon VPg addition was monitored. eIF4E concentration, 360nM. Steady state titration according to(6) HE, His_6eIF4E, HV, His_6VPg.

References

1. Romero, P., Obradovic, Z., Li, X., Garner, E. C., Brown, C. J., and Dunker, A. K. (2001) Sequence complexity of disordered protein. *Proteins Struct. Funct. Bioinforma.* **42**, 38–48
2. Sickmeier, M., Hamilton, J. A., LeGall, T., Vacic, V., Cortese, M. S., Tantos, A., Szabo, B., Tompa, P., Chen, J., Uversky, V. N., Obradovic, Z., and Dunker, A. K. (2007) DisProt: the Database of Disordered Proteins. *Nucleic Acids Res.* **35**, D786–D793
3. Tompa, P. C. N.-R. P. T. 2010 (2010) *Structure and function of intrinscially disordered proteins*, Chapman & Hall/CRC Press, Boca Raton
4. Roudet-Tavert, G., Michon, T., Walter, J., Delaunay, T., Redondo, E., and Le Gall, O. (2007) Central domain of a potyvirus VPg is involved in the interaction with the host translation initiation factor eIF4E and the viral protein HcPro. *J Gen Virol*. **88**, 1029–1033.

5. Xue, B., Dunbrack, R. L., Williams, R. W., Dunker, A. K., and Uversky, V. N. (2010) PONDR-FIT: A meta-predictor of intrinsically disordered amino acids. *Biochim. Biophys. Acta - Proteins Proteomics*. **1804**, 996–1010
6. Michon, T., Estevez, Y., Walter, J., German-Retana, S., and Le Gall, O. (2006) The potyviral virus genome-linked protein VPg forms a ternary complex with the eukaryotic initiation factors eIF4E and eIF4G and reduces eIF4E affinity for a mRNA cap analogue. *Febs J*. **273**, 1312–1322.
